# Characterization of Puerto Rican West Nile Virus isolates in mice

**DOI:** 10.1186/s12985-015-0363-8

**Published:** 2015-09-11

**Authors:** Elba V. Caraballo, Elizabeth Hunsperger, Idalí Martínez

**Affiliations:** Department of Microbiology and Medical Zoology, University of Puerto Rico, Room A-355 UPR-Medical Sciences Campus, PO Box 365067, San Juan, 00936-5067 Puerto Rico; Centers for Disease Control, Division of Vector Borne Diseases, Dengue Branch San Juan, San Juan, Puerto Rico

## Abstract

**Background:**

West Nile virus (WNV) is a neurotropic arbovirus that was first isolated in 1937 in the West Nile District of Uganda. The virus emerged in New York in 1999 and is now endemic in North America (2007). The first virus isolates from Puerto Rico were obtained in 2007 from a chicken (PR20wh) and a mosquito pool (PR423). Our study further characterized these viral isolates using *in vitro* plaque morphology assays and *in vivo* using a Balb/c mice pathogenesis model.

**Methods and results:**

In the *in vitro* experiments, PR WNV isolates produced significantly smaller plaques in Vero cells compared to the New York 1999 strain (NY99). For the *in vivo* experiments, PR WNV isolates were propagated in mammalian (Vero) and insect (C6/36) cell lines and then inoculated in Balb/c mice. When WNV was propagated in Vero cells, we observed a trend towards significance in the survival rate with PR20wh compared to NY99 (log rank, p = 0.092). Regardless of whether the viral isolates were propagated in Vero or C6/36 cells, we found a significantly greater survival in mice infected with PR20wh strain, when compared to NY99 (log rank, p = 0.04), while no statistical difference was detected between PR423 and NY99 (p = 0.84). The average survival time (AST) in mice was significantly lower in C6/36-derived PR423 when compared to C6/36-derived NY99 (*t*-test, p = 0.013), and Vero-derived PR423 (*t*-test, p < 0.001). Eight days post infection in mice the viral load in brain tissue for Vero-derived PR423 was significantly higher when compared to NY99 and PR20wh.

**Conclusions:**

These results suggest that the PR WNV isolate, PR20wh, is a less pathogenic strain in mice than NY99. Moreover, we found that PR423 is a pathogenic isolate that causes faster mortality than NY99, when propagated in C6/36.

## Background

West Nile virus (WNV) is a neurotropic flavivirus which emerged in 1999 in North America causing viral encephalitis primarily in the elderly and immunocompromised individuals [1]. WNV is now endemic in Africa, Asia, Europe and more recently in North America [2–5]. Since its emergence in North America, serological evidence in horses and birds indicated that the virus was widely distributed throughout Mexico [6–9], the Caribbean [10–12], Central America [13] and South America, specifically Colombia, Venezuela, and Argentina [14–17]. Although the evidence of WNV circulation in Brazil is limited to detection of WNV-specific neutralizing antibodies in horses [15, 18, 19], a human case of WNV encephalitis was recently reported [20], suggesting the emergence of WNV in this country.

WNV disease in humans has been rarely reported in Latin America compared to reports from North America. Additionally the high pathogenicity in American crows (*Corvus brachyrhynchos*) and house sparrows (*Passer domesticus),* exhibited by the original isolate from 1999 (NY99 strain) and the subsequently evolved strain from 2002 (WN02) has not been observed throughout the Caribbean, Central and South America. A possible explanation for these differences in pathogenesis between North America and Latin America viral strains is the evolution of less virulent strains in Latin America. Alternatively, cross-protective immunity in humans due to the presence of other flaviviruses in the Caribbean and Latin American countries could partially explain the paucity in the number of reported human cases with WNV disease in this region.

In Puerto Rico (PR), WNV was isolated in 2007 from sentinel chicken serum (PR20wh) and *Culex nigripalpus* mosquito pool (PR423), representing the first isolates from the Caribbean [12, 21]. Serological evidence in a sentinel chicken surveillance indicated that the emergence of WNV in PR occurred in June of 2007 [12]. Sequence analysis of the prM and E viral genes determined one amino acid difference (V159A) between PR 2007 strains and NY99 [21]. This mutation was also observed in the current dominant clade (WN02) circulating in the United States, associated with increased viremia [22]. Interestingly, human disease was rarely detected following the introduction of WNV in PR; only one symptomatic WNV case was identified in an enhanced surveillance study in 2007 [23].

In order to better understand the lack of both animal and human WNV disease in PR following its introduction in 2007, pathogenesis studies in mice were performed with PR isolates (PR423 and PR20wh). Comparisons of mouse survival rates, viremia levels and viral load in brain tissue between NY99 and PR isolates (PR423 and PR20wh) were evaluated. The results of our study showed that PR20wh was less pathogenic in mice than NY99 while PR423 caused similar pathogenicity with faster mortality than NY99.

## Results

### Plaque morphology and growth curve analysis

**Fig. 1:**
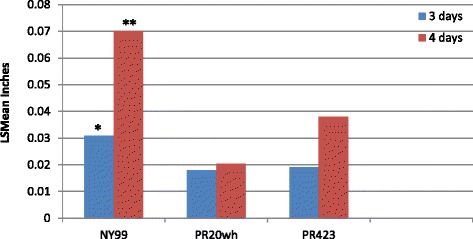
Plaque size for PR isolates compared to its parental strain, NY99. Vero cells were infected for either 3 or 4 days with the WNV and plaque sizes were measured expressed as least square means (LS Means) in inches. ANOVA analysis indicated that the PR isolates had statistically significantly smaller plaques than NY99. (*p = 0.002, **p = 0.0001)

Vero cells were infected for 3 and 4 days with WNV NY99, PR20wh and PR423, and plaque size was measured. PR isolates showed smaller plaques when compared to its parental strain NY99 (Fig. 1). In addition, only a slight difference in plaque size from day 3 to day 4 was observed in the PR20wh isolate. ANOVA analysis indicated significant differences in plaque size between NY99 and PR isolates (*p* = 0.002 (day 3), *p* = 0.0001 (day 4)).

### Pathogenesis of WNV strains in mice

**Fig. 2:**
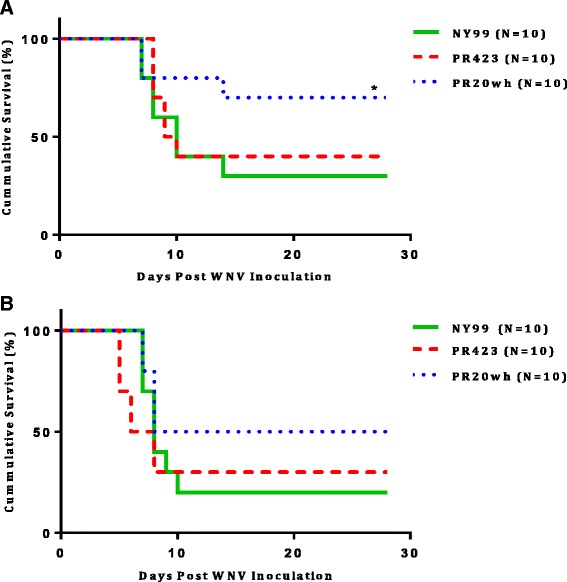
Survival after WNV inoculation. Nine week old Balb/c mice were IP inoculated with 10^5^ pfu of each WNV isolate, collected from either Vero (**a**) or C6/36 (**b**) cells. For each group, 10 mice were observed for morbidity and mortality for 28 days and the other 20 mice were sacrificed on days 2, 4, 6, 8, and 10 post-infection (4mice/day). Log rank test was performed to determine differences in survival rate between the PR isolates and the parental strain, NY99. When strains were Vero-derived, a trend towards a higher survival was observed in mice inoculated PR20wh when compared to NY99 (*p = 0.092). No significant differences in survival were observed, when mice were inoculated with C6/36-derived viruses. Only one experiment was performed

Mice were inoculated by intraperitoneal (IP) injection with 10^5^ plaque forming units (pfu). This is the median viral dose of inoculum recommended *in vivo* to achieve a similar dose as inoculated by *Culex* mosquito species from a natural infection [24]. Most infected mice presented clinical signs of WNV disease such as, weight loss, ruffling coat, hunchback posture, and hind leg paralysis by 6 days post infection (dpi). For mice inoculated with the Vero cell–derived viruses from NY99, PR423 and PR20wh, we observed a morbidity of 90, 80 and 80 %, respectively. Similar morbidity and convalescence results were observed in mice inoculated with C6/36 derived WNV when compared with the Vero derived viruses. Mortality in mice inoculated with the Vero derived NY99 and PR20wh was observed starting on 7 dpi, followed by the PR423 group on 8 dpi. The mice survival rate from the Vero derived WNV strains were 30 % for NY99, 40 % for PR423 and 70 % for PR20wh (Fig. 2a). A trend towards a higher survival rate in PR20wh-inoculated mice was observed when compared to the control group, NY99 (*p* = 0.092). However, no significant differences in survival were observed between PR423 and NY99. Mortality of mice inoculated with the C6/36 derived PR423 isolate began on 5 dpi, followed by the other two groups (NY99 and PR20wh) on 7 dpi. Survival rate in mice of the C6/36-derived WNV was 20 % for NY99, 30 % for PR423 and 50 % PR20wh (Fig. 2b). Statistical analysis determined no significant differences in survival rates between the three viruses (NY99, PR423 and PR20wh) derived from C6/36 cells.

**Fig. 3:**
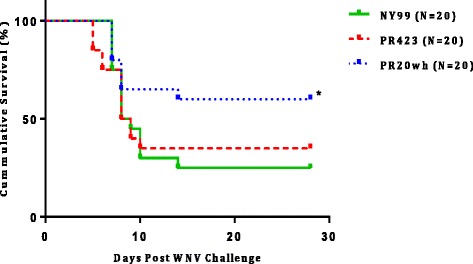
Overall survival after WNV inoculation. Survival experiments from Vero and C6/36 cell derived WNV were pooled. Log rank test demonstrated that PR20wh caused higher survival in mice than NY99 (*p = 0.04)

The comparison of the survival rate was performed for each WNV strain depending on the cell type used for virus propagation (Vero vs. C6/36). Survival rate for all groups infected with C6/36-derived virus decreased by at least 10 %, in comparison with the groups inoculated with Vero-derived WNV. However, the differences in survival rate by viral source were not statistically significant. Therefore, we combined the results from the two experiments and compared the survival rate of the 3 viruses regardless of the viral source. The overall survival rate after WNV inoculation was 25 % for NY99, 35 % for PR423 and 60 % for PR20wh (Fig. 3). The survival rate for the mice inoculated with PR20wh isolate was significantly higher (*p* = 0.04) when compared to NY99. These results suggest that PR20wh displayed an attenuated phenotype in 9-week-old BALB/c mice at the inoculum of 10^5^ pfu.

### Average survival time after WNV inoculation

Table 1 shows the average survival time (AST) calculated following inoculation with WNV isolates. A higher mortality rate was observed in mice when inoculated with the PR423, derived either from Vero cells (AST = 8.66 ± 0.81) or from C6/36 cells (AST = 6.14 ± 1.34). However PR423 isolate didn’t cause higher mortality compared to NY99. Statistical analysis showed significantly lower AST in PR423-inoculated mice than in NY99-inoculated mice, when both viruses were C6/36-derived (*t*-test, *p* = 0.013). Comparison of the AST of PR423 propagated in C6/36 vs. Vero cells, also revealed a significant difference (*t*-test, *p* = <0.001). These results suggested that C6/36-derived PR423 caused faster mortality than C6/36-derived NY99 and Vero-derived PR423.

**Table 1:** Average survival time following inoculation with WNV

WNV Strains	Vero derived	C6/36 derived
	AST ± SD	AST ± SD
NY99	9.14 ± 2.47	8 ± 1.06*
PR423	8.66 ± 0.81**	6.14 ± 1.34*
PR20wh	9.33 ± 4.04	7.6 ± 0.54

This table shows the average survival time (AST) ± standard deviation of Balb/c mice after inoculation with the WNV NY99, PR423 and PR20wh. The student *t*-test analysis showed a significantly lower AST in the C6/36-derived PR423 group when compared to C6/36-derived NY99 (p = 0.013*) and Vero-derived PR423 strains (*p* < 0.001**)

*AST* average survival time, *SD* standard deviation, PR423 and PR20wh, Puerto Rico WNV isolatesWNV = West Nile virus

Student *t* test: PR423 (C6/36) VS:PR423 (Vero)** = *p* < 0.001, Student *t* test: PR423 (C6/36) VS:NY99 (C6/36)* = *p* = 0.013

### Comparison of WNV PR and NY99 Viremia in Mice

**Fig. 4:**
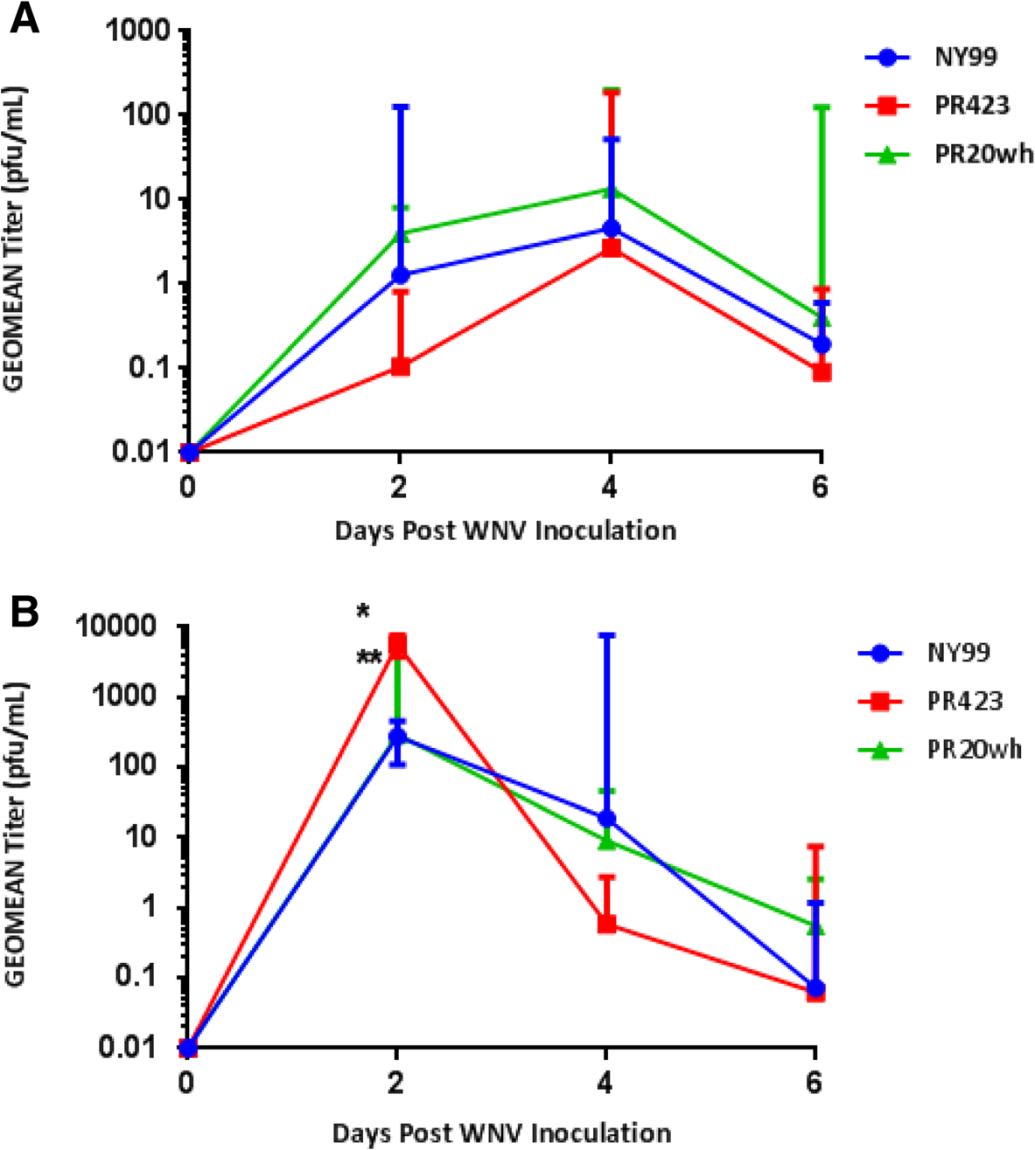
Viremia levels after WNV inoculation. Viremia levels of mice inoculated with Vero–derived (**a**) and C6/36-derived (**b**) strains. An ANOVA analysis showed significantly higher peak viremia in mice inoculated with C6/36-derived PR423 isolate when compared to the other groups (*p < 0.001 PR423 vs. NY99). Viremia levels of PR423 on day 2 were also significantly different when compared according to the cell source (**p < 0.0001 PR423-Vero vs. PR423-C6/36 on day 2)

Low levels of viremia (>10 pfu/ml) were observed for all Vero-derived WNV strains with the peak viremia detected on 4 days post inoculation (dpi) (Fig. 4a). No statistical differences in viremia levels were noted among these three viruses. For C6/36-derived viruses, the peak viremia was detected on 2 dpi (Fig. 4b). When comparing the peak viremia of the PR isolates to NY99, we found a significantly higher viral titer in mice inoculated with PR423, when the virus was propagated in C6/36 cells (*p* ≥ .001). Moreover, significant differences were observed on viremia levels for **Vero-derived PR423 on 2dpi by cell type (**p ≥ .0001; PR423-Vero vs. PR423-C6/36). These results suggest that PR423 was associated with high viremia levels when the virus was propagated in C6/36 cells.

### Comparison of WNV PR and NY99 Viral load in mouse brain

**Fig. 5:**
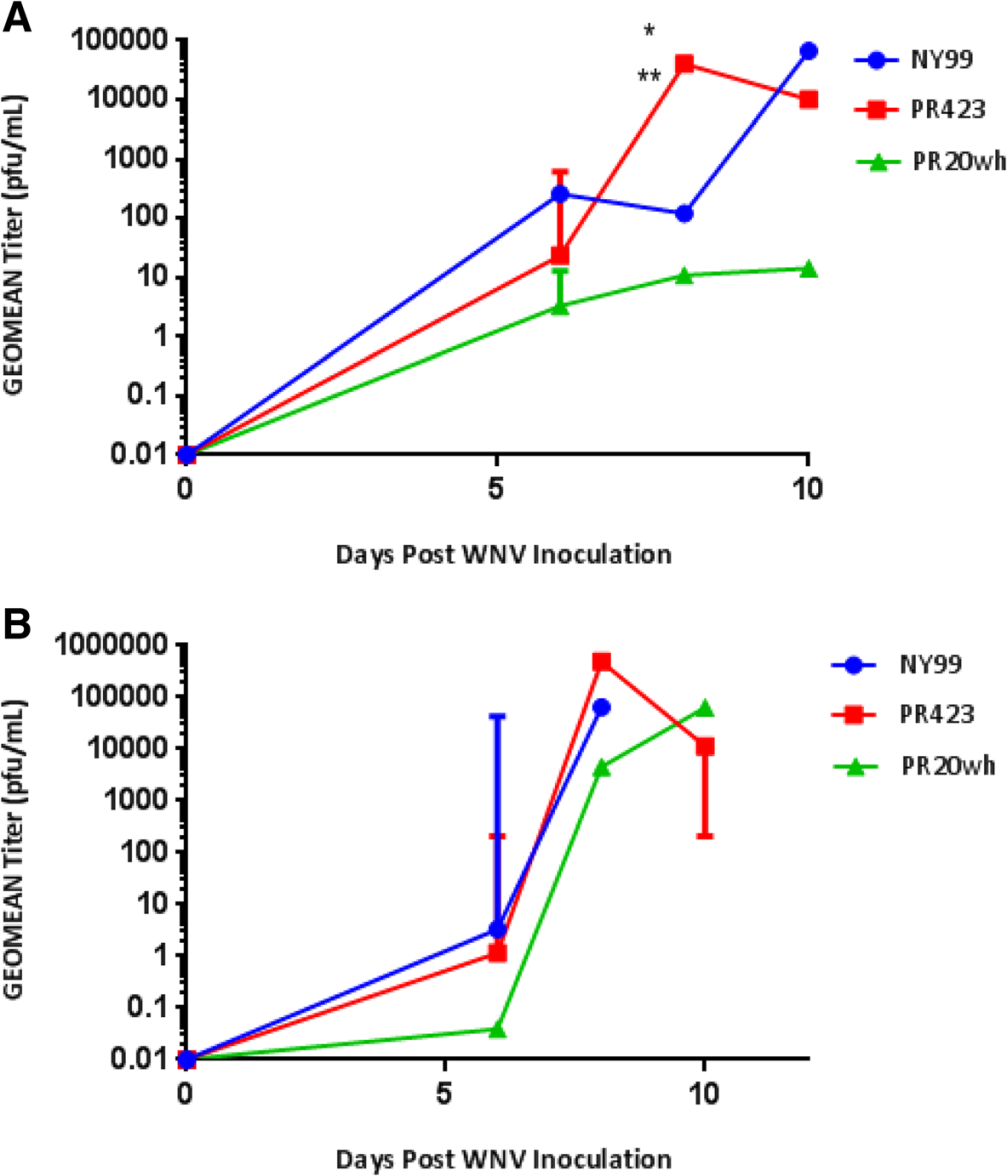
Viral Load after WNV inoculation. Viral load of mice inoculated with Vero–derived WNV (**a**) and C6/36-derived WNV (**b**) isolates. One-way ANOVA analysis showed that viral load for Vero-derived PR423 isolate on 8dpi was significantly higher when compared to NY99 and PR20wh (*p < 0.001 PR423 vs. NY99 and **p = 0.0001 PR423 vs. PR20wh)

The viral titer in brain specimens peaked on 8dpi only for PR423-inoculated group (Fig. 5). Viral titer for Vero-derived PR423 was significantly higher compared to NY99 and PR20wh on 8 dpi (Fig. 5a). C6/36-derived PR423 inoculated mice displayed higher but not significantly different levels of viral titer on 8 dpi, when compared to NY99 and PR20wh (Fig. 5b) [18]. The PR20wh isolate caused the lowest viral titer in brain specimens when derived from Vero cells (Fig. 5a). However, this isolate exhibited an overall higher viral titer in brain specimens when grown in C6/36 cells, although this difference was not statistically significant (Fig. 5b). Viral titers in brain specimens for PR20wh corresponded with the higher survival rate, since the lowest viral titer and highest survival rate was observed when the virus was Vero-derived. Moreover, C6/36-derived PR20wh caused an increase in viral titer and a decrease in survival rate.

## Discussion

The main objective of our study was to characterize the PR WNV isolates in mice and determine if pathogenesis was dependent on virus propagated in mosquito or mammalian cells. Previous studies suggested that infection with WNV strains passaged in mosquito cells (C6/36) would cause higher mortality in mice than those passaged in Vero cells [25–28]. Plaque morphology comparisons between the PR isolates and NY99 indicated that PR isolates produced significantly smaller plaques suggestive of a less pathogenic virus [29, 30]. However after day 4 of infection, the plaque size suggested that the two PR isolates were different since plaque size continued to increase in PR423 infected cells over time but little or no change was observed in plaque size from PR20wh isolate.

The cell source (mammalian vs insect) used for virus propagation plays an important role in the initial virus–host interactions. Several *in vitro* studies with WNV and alphaviruses demonstrated a preferential binding of mosquito cell-derived viruses to the dendritic cell-intracellular adhesion molecule (ICAM)-3 grabbing non integrin related (DC-SIGNR) and an enhanced infection of dendritic cells with mosquito cell-derived viruses compared to viruses propagated in mammalian cells [25–28]. Although *in vitro* studies demonstrated the importance of cell source in the pathogenesis of WNV, limited information is available *in vivo* [25]. Lim et al., examined WNV pathogenesis in mice when virus was derived from mosquito versus mammalian cells and concluded that cell source caused a minimal effect in the mortality of the infected mice at low viral doses (1 and 10 pfu) [31]. Our results were consistent with Lim et al., findings as no significant differences in survival were observed when NY99 and the PR isolates when derived from either mosquito or mammalian cells. When the results from both *in vivo* experiments were combined for analysis, the overall survival rate was significantly higher in mice inoculated with PR20wh in comparison to those inoculated with NY99. These data, in addition to the observed small plaque morphology for PR20wh, suggested that this isolate is less pathogenic in mice than NY99. Similar results were obtained in studies from other Latin America WNV isolates [14, 32, 33]. Indeed, WNV isolate from Tabasco, Mexico (TM171-03) when challenged in American crows, house sparrows and a murine model was shown to be attenuated [9, 29, 32]. Likewise, a variant of the WN02 strain isolated in Houston, Texas (BIRD1153) also showed reduced neuroinvasiveness in mice [30, 32, 34]. Higher survival rates and small plaque morphology are characteristics of the Mexican attenuated WNV variant TM171-03, similar to the findings observed with PR isolate, PR20wh [9, 29, 30, 32].

In the *in vivo* experiments, the peak viremia was significantly higher for PR423 in comparison to NY99 when the virus was propagated in C6/36 cells. This was not surprising since PR423 has the same V159A mutation as the WNV02 dominant clade which correlates with higher viremia [12, 35]. Most pathogenesis studies have shown that WNV-induced mortality in mice correlates with WNV neuroinvasion [36]. In accordance to this observation, the peak viral titer observed in brain specimens was detected earlier (8 dpi) in the PR423 infected groups while viral titers continued to increase up to 10dpi in the NY99 and PR20wh infected groups. This may explain the AST results suggesting that PR423 causes faster mortality in mice than NY99. We also observed a direct relationship between virus titers in brain specimens and mortality in the PR20wh-inoculated groups. When PR20wh was propagated in Vero cells, the lowest viral titer in brain specimens was observed along with higher survival. The result from our study was consistent with the attenuated Mexican variant (TM171-03 pp1) where reduced neuroinvasion in a murine model was observed [12, 29, 35].

Our findings showed that WNV isolates from PR exhibited two different phenotypes, PR20wh exhibited a less pathogenic phenotype than NY99 while PR423 had similar pathogenicity to its parental strain. In Colombia a similar situation was observed where a pathogenic phenotype which exhibited high virulence in Balb/c mice was observed from two WNV isolates from Colombia (COL 524/08, 9835/08) originally obtained from healthy captive American Flamingoes *(Phoenicopterus ruber)* [37]. However, two strains recently isolated from mosquito pools in Colombia were reported to be closely related to attenuated strains from Texas [38]. Although phenotypic characterization of these new Colombian strains has not yet been perform to confirm attenuation, Lopez et al., findings demonstrated the circulation in Colombia of genetic diverse WNV strains [38]. Thus, it is possible that WNV strains isolated from both the Caribbean and Latin America have variable pathogenic capacities.

A large sample size of North America isolates have been collected and sequenced since the introduction of WNV in 1999, due to improved surveillance and the high pathogenicity of these strains in horses and bird species [32]. Such high pathogenicity has not been observed in Latin American and the Caribbean WNV strains [32]. Moreover, there is paucity in the number of reported human cases with WNV disease in Latin America and the Caribbean [39]. Indeed, only one symptomatic case of WNV disease was identified in PR during an enhanced human surveillance study on 2007 [23] and recently a human case of WNV encephalitis was reported in Brazil (Vieira et al., [20]). Possible explanations for the differences in WNV disease observed in humans, horses and birds between North America compared virus strains and Latin America include: 1. The circulation of attenuated strains in Latin America and the Caribbean, 2. the natural resistance to WNV infection in birds in Latin America [40] 3. an increased avian diversity in the tropics [41] 4. the co-circulation of other flaviviruses such as dengue virus that may confer some cross-protective immunity in humans and 5. the limited viral isolates obtained due to non-sustainable WNV surveillance in humans and animals in Latin America [42]. Additional studies are needed to better understand the genotypes and their corresponding phenotypes of WNV strains circulating in PR and Latin America.

## Conclusions

PR423 and PR20wh are the first WNV isolates from the Caribbean. PR20wh was less pathogenic than NY99 whereas PR423 had similar pathogenesis as its parental strain. Further characterization at the molecular level is necessary to understand the difference in pathogenicity of these two WNV viral strains.

## Methods

### Virus strains

WNV isolates were obtained from the reference collection from the Centers for Disease Control and Prevention, Division of Vector-Borne Diseases, Dengue Branch, San Juan, Puerto Rico (CDC DVBD-DB). The NY99 strain was isolated from an infected Chilean flamingo in the Bronx Zoo and was passaged once in suckling mice and twice in Vero cells [43]. In this study, NY99 was used at passage 4. The Puerto Rico isolates, PR423 and PR20wh, were isolated from mosquito pools (*Culex nigripalpus*) and serum from a sentinel chicken, respectively. These viral strains were passaged twice in Vero (ATCC) cells prior to this study [12]. In this study the PR strains were used at passage 3.

### Virus infection

C6/36 (*Aedes albopictus* cell line ATCC® CRL-1660) and Vero (African green monkey kidney cells) were infected with WNV (NY99, PR423, and PR20wh) at a multiplicity of infection (MOI) of 0.1 as previously described [43]. Briefly, C6/36 and Vero cells monolayers were infected and incubated for one hour at room temperature. C6/36 cells were maintained in DMEM supplemented with 10 % Fetal Bovine Serum (FBS) and incubated at 33 °C for 3 days without CO_2_. Vero cells were maintained in M-199 supplemented with 10 % FBS and incubated at 37 °C for 3 days. Viral stocks were collected for virus titration. The envelope protein glycosylation pattern of the PR isolates and NY99 was verified by western blood analysis.

### Virus plaque titration assay

Vero cells were seeded in Costar 12-well cell culture plates (Corning Incorporated, Corning, NY) and incubated for 24 h (hrs) at 37 °C in 10 % CO_2._ The cell monolayers were inoculated with 10-fold serial dilutions of each viral strain; in a final volume of 0.1 milliliter (ml). Viral adsorption was performed for 1 h at 37 °C with rocking of the plates every 15 min. A 2-ml overlay of MEM, 5 % FBS, and 0.6 % agarose was added after incubation as described by Eckels et al. [44]. The infected monolayers were incubated at 37 °C in 5 % CO_2_. After 2–3 days of infection, a second overlay containing 1.5 % neutral red (Sigma–Aldrich Co., St. Louis, MO), was added to each well, and the plates were incubated at 37 °C in 5 % CO_2_ overnight. The following day the plaques were counted and the viral titer was expressed as pfu/ml.

### Plaque size analysis

Vero cells were seeded into 6-well plates and infected with the PR isolates and NY99 (all viral stocks were previously propagated in Vero cells) for 1 h. A 2-ml overlay of MEM, 5 % FBS, and 1 % SeaKem ME AGAROSE (Lonza Rockland, ME) was added following virus adsorption as previously described by Eckels et al. [44]. After staining with neutral red, plaques were counted at the indicated times post-infection. Plaque size analysis was assessed using calipers to measure twenty plaques in two separate wells on day 3 or 4 days post-infection (dpi).

### Animal studies

Nine week old Balb/c mice were purchased from Charles River Laboratories (Boston, MA), and were intraperitoneally (IP) inoculated with each WNV strain collected from either mosquito (C6/36) or mammalian cells (Vero) as previously described by Ben-Nathan et al., [45]. Mice were divided in groups of 30, of which 10 mice were observed for morbidity and mortality for 28 days and the other 20 mice were sacrificed on days 2, 4, 6, 8, and 10 post-challenge for viral load determinations. Symptoms of WNV encephalitis such as weight loss, ruffling, hunchback posture and hind-leg paralysis were recorded. Blood specimens were obtained by retro-orbital method on the day of WNV inoculation and intracardially from sacrificed mice [46]. The brain was removed from sacrificed animals. The average survival time (AST) (the average time in days needed to cause death of mice) excluding survivors, was determined. Real time RT-PCR was performed on serum and brain specimens to measure viremia and viral load, respectively. Mice that survived the WNV infection were sacrificed at the end of the experiment (28 days post-infection), and intra-cardiac and brain samples were obtained. Animal procedures were performed in compliance with the University of Puerto Rico Medical Sciences Campus Institution Animal Care and Use Committee (IACUC), approved protocol 2460408.

### Viral RNA isolation

Viremia and viral load was determined by real-time quantitative RT-PCR from serum samples and brain specimens collected on 0, 2, 4, 6, 8 and 10 days post WNV infection. The QIAmp viral RNA kit (QIAgen, Valencia, CA) was used for viral RNA isolation from serum samples and from virus stocks (for the standard curve). Briefly, 560 μl of the Buffer AVL were added to 100 μl of the serum or virus stock and mixed. Following 10 min incubation at room temperature, 560 μl of ethanol (96–100 %) was added. Next, 630 μl of the sample was applied to the QIAamp Mini spin column and centrifuged at 8000 rpm for 1 min. Then 500 μl of Buffer AW1 was added to the column and centrifuged for 1 min at 8000 rpm, followed by 500 μl of Buffer AW2 and a second centrifugation for 3 min at 14,000 rpm. The column was placed in a clean 1.5 micro centrifuge tube and 60 μl of Buffer AVE was added and incubated for 1 min at room temperature. Elution of the RNA was completed following a centrifugation for 1 min at 8000 rpm. Viral RNA samples were stored at −70 °C until use.

Viral RNA extraction from the brain tissue was performed using the QIAgen RNeasy Lipid Tissue Midi Kit. The brain tissue was harvested in RNAlater RNA Stabilization Reagent (QIAgen, Valencia, CA) and stored at −80 °C until processing. Briefly, 300 to 310 mg of brain tissue was homogenized in 5 mL of QIAzol reagent (QIAgen, Valencia, CA) using 2 ml tissue grinders (Wheaton, Millville, NJ). After homogenization, samples were transferred into 15 ml polypropylene tubes and mixed with 1 mL of chloroform. After 3 min incubation at room temperature, samples were centrifuged for 20 min at 3000 rpm at 4 °C. Then, the upper aqueous phase was transferred to a new polypropylene tube, and 3 ml of 70 % ethanol were added. Samples (4 mL) were transferred to a column centrifuged for 5 min at 3000 rpm at room temperature and the flow-through was discarded. This procedure was repeated with the remaining sample. The sample was washed once with 4 ml of buffer RW1, (2 min centrifugation at 3000 rpm) and twice with 2.5 ml Buffer RPE. A 5 min centrifugation at 3000 rpm was performed and the column was transferred into a new 15 ml tube. Elution was performed with 150ul RNase-free water. After 2 min incubation at room temperature, the sample was centrifuged for 3 min at 3000 rpm, for elution. The viral RNA samples were stored at −70 °C until use.

### Real time RT-PCR

TaqMan assays were performed to determine WNV viral load as previously described by Lanciotti and collaborators [47]. Briefly, a master mix consisting of 5 μl of viral RNA (vRNA) extracted from serum or brain samples, 50pmol of each primer and 10pmol of the FAM labeled probe was prepared and adjusted to a total of 50uL. The iScript One-Step RT-PCR kit for Probes (Bio-Rad Hercules, CA) was used. Serial dilutions of the viral RNA extracted from WNV-NY99 stocks were made to construct the standard curve and determine the pfu/ml or pfu/ mg based on cycle threshold values (Ct). Amplification protocol was performed as follows: 1 RT cycle of 50 °C for 30 min, 1denaturation cycle at 95 °C for 10 min, 45 annealing cycles at 95 °C for 15 s and 60 °C for 1 min and 1 extension cycle of 68 °C for 3 min using an iQ4 Multicolor Real-Time PCR Detection System (Bio-Rad, Hercules, CA).

### Statistical analysis

Survival analysis was performed by the Kaplan-Meier method using log-rank test to compare survival rates among groups. Average survival time (AST), was calculated for every group of inoculated mice and compared using student *t* test. Viral loads were log transformed for statistical tests. Mean log viral load was analyzed by one way ANOVA and the geometric mean titers were analyzed by one-way ANOVA and the Bonferroni adjustment was applied for multiple comparisons. All analysis was performed using Graphpad® Prism statistical program, Version 5 (La Joya, CA).

## Abbreviations

AMCRs: American crows
AST: Average survival time
ATCC: American Type Culture Collection
CDC: Centers for Disease Control and Prevention
Ct: Cycle threshold values
COL: Colombia
DB: Dengue Branch
DC-SIGNR: Dendritic cell-intracellular adhesion molecule (ICAM)-3 grabbing non integrin related
DMEM: Dulbecco’s modified Eagle medium
dpi: Days post infection
vRNA: viral ribonucleic acid
DVBD: Division of Vector-Borne Diseases
E: Envelope
FBS: Fetal bovine serum
HOSPs: House sparrows
IACUC: Institution Animal Care and Use Committee
IP: Intraperitoneally
LS Means: Least Squares Means
MEM: Minimum essential medium
MOI: Multiplicity of Infection
NY99: New York 99
PBS: Phosphate buffered saline
PFU: Plaque forming unit
PR20wh: Puerto Rico 20wh
PR423: Puerto Rico 423
PRDH: Puerto Rico Department of Health
RT-PCR: Real Time- Polymerase Chain Reaction
WNV: West Nile virus
